# Survival of Cancer Patients with Co-Morbid Tuberculosis in Thailand

**DOI:** 10.31557/APJCP.2021.22.8.2701

**Published:** 2021-08

**Authors:** Pallop Siewchaisakul, Sirinya Nanthanangkul, Chalongpon Santong, Krittika Suwanrungruang, Patravoot Vatanasapt

**Affiliations:** 1 *Faculty of Public Health, Chiang Mai University, Thailand. *; 2 *ASEAN Cancer Epidemiology and Prevention Research Group, Khon Kaen, Thailand. *; 3 *Udonthani Cancer Hospital, Medical Service Department, Ministry of Public Health, Thailand. *; 4 *Cancer Unit, Srinagarind Hospital, Faculty of Medicine, Khon Kaen University, Khon Kaen, Thailand. *; 5 *Department of Otorhinolaryngology, Faculty of Medicine, Khon Kaen University, Khon Kaen, Thailand. *

**Keywords:** Cancer survival, comorbidity tuberculosis, Thailand

## Abstract

**Background and objective:**

We aimed to investigate the survival time and its related factors among cancer patients with co-morbid tuberculosis (TB) in Thailand.

**Methods:**

We conducted this retro-prospective cohort study on cancer patients without co-morbid TB using the data from population–based cancer registry of Khon Kaen, TB databases from the Khon Kaen Central Hospital, and the Region 7 Office of Disease Prevention and Control from 2001 to 2015 to determine the onset of TB after cancer. The cancer patients were then followed up until 2017 to assess their survival status. The Kaplan-Meier method, log-rank test, and Cox proportional hazard regression were used to estimate cumulative survival curves, compare various survival distributions, and adjusted hazard ratios.

**Results:**

Lung, head and neck, and liver cancers led to a significantly different survival time between patients with and without co-morbid TB. After adjustment, it was found that patients suffering from lung, head and neck, or liver cancer and co-morbid TB had significantly lower risk of death than those without co-morbid TB. Based on the stratified analysis, lung cancer patients with distant metastasis and co-morbid TB had 3.01-fold and 2.99-fold significantly increased risk of death compared to those without co-morbid TB.

**Conclusion:**

We found that cancer patients with co-morbid TB were at lower risk of death compared with those without co-morbid TB. In addition to cancer stage, it seems that cancer comorbidity with TB could modify the risk of death for lung cancer patients.There is a need for further studies to support our findings including other related risk factors.

## Introduction

Tuberculosis (TB) remains the most common infectious disease with high mortality worldwide (Cohen et al., 2019; Shu et al., 2019). According to the global TB report published by World Health Organization (WHO), approximately 10.0 million people were infected with TB and 1.3 million death every year due to this disease. Geographically, most people (44%)who developed TB in 2019 were in the WHO South-East Asia (SEA). Thailand, a country located in the SEA, has been placed among the top 30 countries with high TB burden. TB incidence in Thailand was 150 per 100,000 and its mortality was 14 per 100,000 in 2019 (Global Tuberculosis Report, n.d.).

Although TB is a communicable disease, the relationship between TB and non-communicable diseases, especially cancer, has been proved in previous studies. According to previous studies, TB increased the risk of lung cancer and vice versa (Shieh et al., 2012; Shu et al., 2019). Using population-based data, a recent study in Thailand revealed that various types of cancer (i.e. lung, lymphoma, hematopoietic, and gastrointestinal cancers) were significantly associated with an increased risk of acquiring TB (Nanthanangkul et al., 2020). 

On the other hand, the mortality rate of cancer patients diagnosed with TB is different depending on the type of the cancer. For instance, a higher mortality rate was detected in patients with respiratory tract cancer and hematology malignancy (30.15% at 12 months and 22.76% at 12 months, respectively) . Interestingly, the overall cancer patients without TB had higher all-cause mortality compared with cancer patient with TB (Shu et al., 2019); however, Shieh et al., (2012) found that lung cancer patient with TB had elevated risk of mortality and had lower survival rate compared with lung cancer patients without TB.

This controversy was also found between lung cancer patients with and without TB regarding survival time. Chai and Shi (2020) showed that there was no different between these groups of patients in terms of survival time. However, Shu, et al., unveiled that lung cancer patients with TB had significantly lower survival time than lung cancer patients without TB (Shu et al., 2019). The difference in survival time is probably due to clinicopathological factors, including tumor site, stage of the disease, histological type, histological grading, type of treatment, and site of metastasis (Siewchaisakul et al., 2016). Albeit, to our knowledge, there are few studies on survival time, especially in Thailand, using population-based data and taking into account clinicopathological factors . Furthermore, most of these studies just focused on lung cancer. Therefore, we aimed to compare survival time between cancer patients with and without co-morbid TB and investigate factors associated with survival time in Thai patients.

## Materials and Methods

*Study design and data collection*

To conduct this retro-prospective cohort study, we obtained our patients’ data from Khon Kaen population–based cancer registry, Khon Kaen Central Hospital, and Office of Disease Prevention and Control Region 7. In brief, we identified cancer patients registered into the Khon Kaen population–based cancer registry between January 1, 2001 and December 31, 2015 according to International Classification of Diseases for Oncology (ICD–O 3rd edition, code C00.0–C80.9). Totally, there were 41,045 cancer patients. TB cases registered between January 1, 2001 and December 31, 2017 were identified based on ICD–10 version 2016, code A15.0–A19.9. After exclusion of duplicates, 26,945 TB cases remained.

We linked databases of cancers and TB via national ID number during the time period of 2001 to 2015, and followed up the patients until 2017 to assess the survival status of each patient by monitoring their medical records and contacting with the death registry of the Thai national statistics database. Finally, 472 cancer patients with co-morbid TB remained.

We included patients’ TB diagnosis, sex, age, primary cancer sites, cancer stage, and cancer treatment as explanatory variables. Data on patients’ death was regarded as response variable. Cancer site was classified into 17 sites in our study comprising of breast, colorectal, liver (include bile duct), lung (with or without histopathological confirmation), lymphoma, hematopoietic (leukemia), female organ (cervix, corpus, uterus, ovary, perineum), male organ (penis, prostate, testis), head and neck (oral cavity, oropharynx, noses and paranasal sinuses, larynx, and hypopharynx) skin, thyroid, digestive organ, brain, urinary tract, unknown primary and other cancers. Due to existence of multiple cancer sites in our patients, we adopted SEER Summary Staging 2000 to categorized the stages of malignant tumors, including localized, regional, distant, not applicable, or unknown. Treatments were grouped into 8 categories as no treatment, only surgery (S), only chemotherapy (C), only radiotherapy (R), S+C, S+R, C+R, and S+C+R. The study was approved by the Ethics Committee of Khon Kaen University (reference number HE 611542).

*Statistical analysis*

Patients’ demographic data were reported as numbers and percentages. Observed survival rates were calculated from a Kaplan-Meier survival curve, and the rates were presented as percentages of patients who were alive after a certain period of time with 95% confidence interval (95% CIs). We performed the log-rank test to identify any significant differences regarding survival time. Those cancers that led to a significant difference with respect to survival time between patients with and without TB were then selected to undergo further analysis. We conducted stratified analysis for identifying possible of modification effect for selected cancers. The associations between survival time and various prognostic factors were analyzed using Cox proportional hazard regression models. The results were reported in terms of crude and adjusted hazard ratios (aHR) with 95% confidence intervals (CIs). All analyses were conducted using Stata version 10.0 (Stata Corp LP, 2007). Except for the process of selecting variables to be included in the multivariate analysis, statistical significance was set as p<0.05.

## Results

A total of 40,948 of patients with various cancers were recruited in this study between 2001 and 2015. Among all 17 cancers, 1.2% of patients (472) had cancers with co-morbid TB. After performing log-rank test, three cancer types with a significantly different survival time were selected, comprising of 2,656 patients with non-histologic type of lung cancer, 1,253 patients with histologic type of lung cancer, 12,948 liver cancer patients, and 2,376 head and neck cancer patients. Histologic type of lung cancer had the highest TB proportion of 2.1% (57 patients), followed by non-histologic type of lung cancer 1.8% (22 patients), head and neck cancer 1.5% (36 patients), and liver cancer 0.5% (60 patients). Most patients were male, aged over 60 years old, had distant stage (except head and neck cancer patients), and underwent no curative treatment (Table 1).

Findings on survival rate with respect to type of cancer with co-morbid TB are depicted in Table 2. We found that survival rate of cancer patients with co-morbid TB suffering from liver, non-histologic type of lung cancer, and head and neck cancer was higher than those without TB . Interestingly, patients suffering from histologic type of lung cancer with co-morbid TB had lower survival rate than those without co-morbid TB and none of patients could survive three years after diagnosis (22.7% vs 30.5%), respectively. 

Stratified analysis of TB was done by stage of cancer only among lung cancer and head and neck cancer patients, due to scant of patients. We found that patients suffering from distant stage of lung cancer with co-morbid TB had significantly higher risk of death than those without co-morbid TB (Table 3). 

Our results revealed that lung cancer patients with co-morbid TB had lower risk of death compared with those without co-morbid TB, which was statistically significant, especially in those with non-histological type of lung cancer (aHR=0.55; 95%CI: 0.37-0.80). The aHR of patients with histologically confirmed lung cancer was=0.61 (95%CI: 0.30-1.23). Other factors and their aHR on lung cancer patients are also reported in Table 4. Male gender was identified as a risk factor for developing lung cancer. in addition, age > 70 years old was also found as a significant risk factor for lung cancer. We also found that the stage of cancer. i.e. regional and distant, significantly increased the risk of both types of lung cancer. It was also found that the magnitude of effect was higher at the distant stage. Patients who did not undergo any treatments showed significantly greater risk of death than patients with histologically confirmed lung cancer who were treated with surgery. Our interaction term of cancer stage and TB infection shown the greater risk of death in both groups of lungs cancer with distant stage co-morbid with TB compared with without TB lung cancer patient. The aHR of patients at distant stage of lung cancer and those with non-histological confirmed lung cancer who suffered from co-morbid TB was 2.99 (95%CI: 1.17-7.69) and 3.01 (95%CI: 1.63-5.57), respectively. The factors associated with survival of liver and head and neck cancer patients are shown in Supplementary of Table 1. 

The survival curves of non-histological type of lung cancer and histological type of lung cancer stratified by cancer stage and TB diagnosis are shown in Supplementary Figures 1 and 2, respectively. There was no distant stage of both lung cancer types patients with TB, that survived over one year.

**Table 1:** Distribution of Demographic and Pathological Characteristics of Cancer Patients

	All cancer		Lung (no-histology)		Lung (histology)		Liver		Head &neck	
Variables	Number (40,948)	%	Number (2,656)	%	Number (1,253)	%	Number (12,948)	%	Number (2,376)	%
TB diagnosis										
TB	472	1.2	57	2.1	22	1.8	60	0.5	36	1.5
Non-TB	40,476	98.8	2,599	97.9	1,231	98.2	12,888	99.5	2,340	98.5
Sex										
Male	21,014	51.3	1,905	71.7	860	68.6	8,895	68.7	1,383	58.2
Female	19,934	48.7	751	28.3	393	31.4	4,053	31.3	993	41.8
Age										
≤ 50	10,789	26.3	328	12.3	242	19.3	1,952	15.1	628	26.4
51-60	9,972	24.4	560	21.1	362	28.9	3,447	26.6	540	22.7
61-70	10,695	26.1	857	32.3	395	31.5	4,108	31.7	554	23.3
>70	9,492	23.2	911	34.3	254	20.3	3,441	26.6	654	27.5
Cancer stage										
Local	2,483	6.1	8	0.3	19	1.5	70	0.5	236	9.9
Regional	5,834	14.2	169	6.4	271	21.6	222	1.7	726	30.6
Distant	8,066	19.7	1,020	38.4	572	45.7	2,655	20.5	420	17.7
Unknown	22,184	54.2	1,456	54.8	387	30.9	9,977	77.1	869	36.6
Na	2,381	5.8	3	0.1	4	0.3	24	0.2	125	5.3
Treatment										
No treatment	22,361	54.6	2,242	84.4	606	48.4	11,537	89.1	830	34.9
Only S	6,685	16.3	18	0.7	55	4.4	836	6.5	395	16.6
Only C	2,942	7.2	161	6.1	340	27.1	345	2.7	162	6.8
Only R	1,841	4.5	178	6.7	113	9	22	0.2	434	18.3
S+C	4,148	10.1	4	0.2	43	3.4	190	1.5	33	1.4
S+R	1,304	3.2	4	0.2	16	1.3	8	0.1	289	12.2
C+R	824	2	45	1.7	70	5.6	6	0.05	185	7.8
S+C+R	843	2.1	4	0.2	10	0.8	4	0.03	48	2

S, Surgery; C, Chemotherapy; R, Radiotherapy

**Figure 1:**
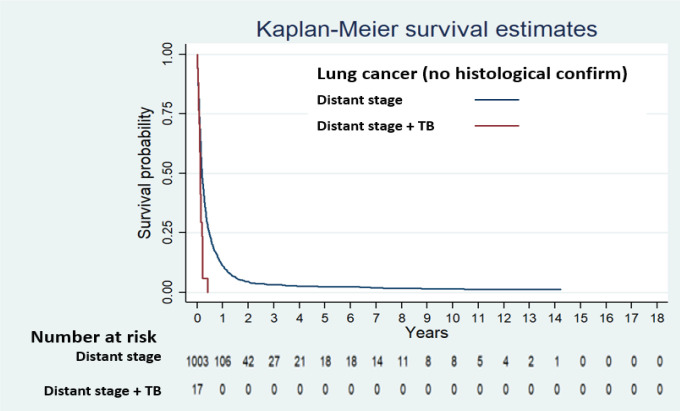
Survival Curve of Distant Lung Cancer (no histological confirm) by TB Diagnosis

**Table 2:** Survival Rate of Lung, Liver, and Head and Neck Cancer Patients with and without TB

Survival time (year)	Survival rate (%)									
	All cancers		Lung (no histological confirm)		Lung (histological confirm)		liver		Head & neck	
	TB (95%CI)	Non-TB (95%CI)	TB	Non-TB (95%CI)	TB (95%CI)	Non-TB (95%CI)	TB (95%CI)	Non-TB (95%CI)	TB (95%CI)	Non-TB (95%CI)
			(95%CI)							
1	61.7	45.7	22.8	16.3	22.7	30.5	55	16.6	80.6	60.2
	(57.1, 65.9)	(45.2, 46.2)	(13.0, 34.3)	(14.9, 17.8)	(8.9, 41.5)	(27.9, 33.1)	(41.6, 66.5)	(16.0, 17.3)	(63.5, 90.2)	(58.2, 62.2)
3	49.8	33.4	19.1 (10.2, 30.3)	8.1	9.1	11	41.5	8.7	63.3	4.3
	(45.2, 54.3)	(32.9, 33.9)		(7.0, 9.2)	(6.1, 25.1)	(9.2, 12.8)	(29.0, 53.6)	(8.2, 9.2)	(45.2, 76.8)	(88.3, 42.3)
5	44.8	29.2	19.1	6.6	-	8	36.1	7	51.4	34.8
	(40.2, 49.3)	(28.7, 29.6)	(10.2, 30.3)	(5.7, 7.7)		(6.5, 9.7)	(24.0, 48.2)	(6.5, 7.4)	(32.7, 67.3)	(32.8, 36.7)
7	39.5	26.7	16	5.9 (5.0, 7.0)	-	7.1	29.9	6	47.5	30.6
	(34.8, 44.1)	(26.3, 27.2)	(7.4, 27.5)			(5.6, 8.8)	(18.6, 42.1)	(5.6, 6.5)	(29.0, 63.9)	(28.6, 32.6)
9	34.9	24.9	16	5.3 (4.4, 6.3)	-	6.4	24.9	5.3	21.1	27.4
	(30.1, 39.8)	(24.5, 25.4)	(7.4, 27.5)			(4.9, 8.2)	(14.2, 37.3)	(4.9, 5.8)	(1.9, 54.3)	(25.4, 29.5)
11	31.1	23.4	16	5.1 (4.2, 6.1)	-	5.9	22.2	4.7	-	25
	(26.0, 36.2)	(23.0, 23.9)	(7.4, 27.5)			(4.4, 7.7)	(11.8, 34.6)	(4.3, 5.2)		(22.9, 27.1)
13	29.7	22.3	16	4.8	-	5.4	22.2	4.5	-	23.4
	(24.6, 35.0)	(21.8, 22.8)	(7.4, 27.5)	(3.9, 5.9)		(3.9, 7.3)	(11.8, 34.6)	(4.1, 5.0)		(21.2, 25.6)
15	25.8	21.5	-	4.5	-	4.7	18.5	4.3	-	21.6
	(19.8, 32.1)	(21.0, 22.1)		(3.5, 5.7)		(2.9, 7.0)	(8.5, 31.4)	(3.9, 4.8)		(19.2, 24.2)
Median survival (year)	2.92	0.74	0.21	0.18	0.37	0.52	1.7	0.19	6.02	1.64
Log-rank test	<0.0001		0.0348		0.5345		<0.0001		0.0487	
(p-value)										

**Table 3:** Stratified Analysis for TB by Stage of Lung and Head and Neck Cancer Patients

Primary cancer	Stage	TB	HR	95%CI	
Lung					
(no histology)					
	Regional				
		No	1		
		Yes	0.62	0.19	2.01
	Distant				
		No	1		
		Yes	2.11	1.3	3.42
Lung (histology)					
	Regional				
		No	1		
		Yes	0.67	0.17	2.7
	Distant				
		No	1		
		Yes	2.4	1.15	4.05
Head & Neck					
	Local				
		No	1		
		Yes	0.36	0.05	2.56
	Regional				
		No	1		
		Yes	1.17	0.65	2.14
	Distant				
		No	1		
		Yes	0.56	0.21	1.51

**Figure 2:**
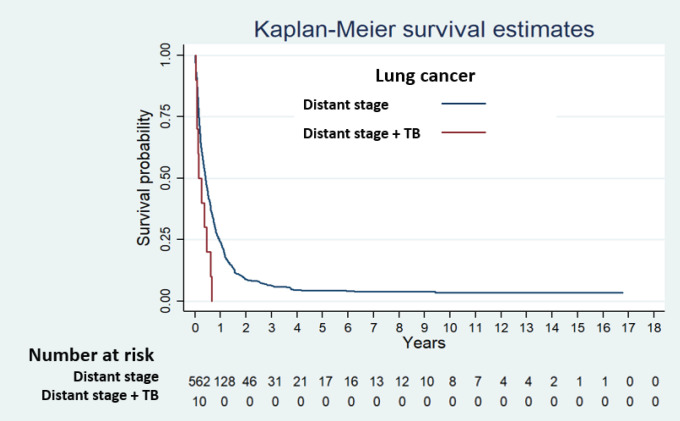
Survival Curve of Distant Stage Lung Cancer by TB Diagnosis

**Table 4:** The Association between TB and Lung Cancers Using Multi-variable Cox Regression

Variables	Lung (no histology)						Lung (histology)					
	Crude HR	95%CI		aHR	95%CI		Crude HR	95%CI		aHR	95%CI	
TB												
No	1			1			1			1		
Yes	0.73	0.55	0.98	0.55	0.37	0.8	1.15	0.74	1.79	0.61	0.3	1.23
Sex												
Female	1			1			1			1		
Male	1	0.92	1.1	1	0.91	1.09	1.06	0.93	1.2	0.99	0.87	1.13
Age												
≤ 50	1			1			1			1		
51-60	0.94	0.81	1.09	0.93	0.8	1.08	1.02	0.85	1.21	0.96	0.8	1.14
61-70	1.05	0.92	1.2	1	0.87	1.14	1.17	0.99	1.39	1.04	0.88	1.24
>70	1.2	1.05	1.37	1.12	0.98	1.28	1.63	1.35	1.96	1.41	1.16	1.71
Cancer stage												
Local	1			1			1			1		
Regional	1.6	0.71	3.64	1.79	0.78	4.08	4.39	2.17	8.89	3.98	1.95	8.14
Distant	2.05	0.92	4.6	2.1	0.93	4.74	6.37	3.16	12.82	4.88	2.4	9.93
Na	1.73	0.43	6.93	2.02	0.5	8.16	3.78	1	14.26	1.97	0.52	7.49
Unknown	1.76	0.79	3.93	1.71	0.76	3.85	4.8	2.38	9.69	3.71	1.82	7.55
Treatment												
Only S	1			1			1			1		
No treatment	0.94	0.58	1.51	0.88	0.54	1.42	3.45	2.48	4.81	2.65	1.89	3.71
Only C	0.57	0.35	0.94	0.53	0.32	0.88	1.75	1.25	2.45	1.37	0.98	1.94
Only R	0.83	0.5	1.36	0.74	0.45	1.23	2.53	1.74	3.68	1.89	1.29	2.76
S+C	0.6	0.2	1.77	0.51	0.17	1.54	0.91	0.57	1.45	0.77	0.48	1.24
S+R	0.62	0.21	1.86	0.52	0.17	1.55	1.42	0.75	2.66	1.02	0.54	1.93
C+R	0.44	0.25	0.78	0.41	0.23	0.73	1.58	1.05	2.36	1.23	0.82	1.85
S+C+R	0.53	0.18	1.57	0.44	0.15	1.32	1.18	0.57	2.43	1.05	0.51	2.18
Interaction terms												
TB (-)	-	-	-	1			-	-	-	1		
Regional with TB (+)	-	-	-	1.33	0.4	4.45	-	-	-	1.21	0.25	5.78
Distant with TB (+)	-	-	-	3.01	1.63	5.57	-	-	-	2.99	1.17	7.69

S, Surgery; C, Chemotherapy; R, Radiotherapy

## Discussion

There are few studies investigating the survival rate of cancers patients with newly diagnosed TB. In addition, the results on survival rate between cancer patients with and without TB are contrasting. Furthermore, to our knowledge, no study conducted in Thailand investigating this issue. Therefore, we investigated survival rate and factors associated with survival of cancer patients with and without co-morbid TB in Thailand. 

We found that only lung, head and neck, and liver cancer led to a significant difference in survival time between cancers patients with and without co-morbid TB. We also found that patients suffering from these cancers and co-morbid TB had significantly less risk of death in comparison with the other group. Notwithstanding, our stratified analysis pointed out that patients at distant stage of histological type of lung cancer and non- histological type of lung cancer had significantly increased risk of death compared with those without TB. 

Although Mycobacterium tuberculosis can cause disease in almost any part of the body, the pathogen is primarily infected in lung (Pai et al., 2016). A previous study has shown that the infection of TB was highest in lung cancer patients compared with patients suffering from other cancers in Thailand (Nanthanangkul et al., 2020). In our study, although lung cancer patients with co-morbid TB showed lower survival time that lung cancer patients without co-morbid TB, the aHR showed that lung cancer patients with co-morbid TB had insignificantly lower risk of death than those without TB. Our result was in line with those revealed by Kuo et al., (2012) concluding that active tuberculosis was an independent predictor of better survival with HR of 0.68 (95% CI: 0.48-0.97). 

One possible explanation for such association in our study could be that the most of lung cancer patients without co-morbid TB (67.42%) were at late stages of the disease (regional and distant stages) compared to lung cancer patients with co-morbid TB (59.10%). With respect to the biological view point, this association can be explained by immune modulation by Interferon-gamma (IFN-γ), a pleiotropic cytokine induced by macrophage via response of T-cell. It plays a major role in coordinating both innate and adaptive immune responses. It is involved in immunological cell signaling and is a critical regulatory protein for overall immune system function (Fenimore and A Young, 2016). The IFN-γ has antiviral, antitumor, and immunomodulatory function and a critical role in recognizing and eliminating pathogens (Schroder et al., 2004; Mendoza et al., 2019). These properties lead to increasing of the phagocytic oxidative burst (Khan et al., 2016). Notably, high dose of IFN-γ can lead to tumor regression (Jorgovanovic et al., 2020). As the result, we hypothesized that the IFN-γ, induced by TB infection in lung cancer, partially contributed to better survival among lung cancer with co-morbid TB infection. 

Withal, Shieh et al., (2012) revealed that lung cancer patients with co-morbid TB had 1.30-fold increased risk of death compared with lung cancer patients without co-morbid TB. Stage of cancer is a well-known prognostic factor for cancer survival, including lung cancer (Maringe et al., 2012; Allemani et al., 2015). Our result on survival time in lung cancer patients with co-morbid TB was inverse.

When we investigated the interaction between TB and cancer stage, we found that patients with distant stage of lung cancer and co-morbid TB had 2.99-fold significant increased risk of death than those without co-morbid TB. 

It is well known that the stage of cancer determines survival and changing the definition of determining stage of lung cancer would led to difference in survival prognosis (Chansky et al., 2017). Some studies demonstrated that reduced IFN-γ in tumor microenvironment and augmented activity of integrin αvβ3 signaling axis brought about metastatic potential of tumor cells. It means the ability of the immune system to fight with TB infection; however, this occurs during receiving immunotherapy treatment (Gong et al., 2008). As aforementioned, cancer stage could modify the effect of TB-infection on hazard of death in lung cancer patients co-morbid with TB. 

The stratified analysis result with respect to stage of head and neck cancer patients was insignificance. Furthermore, we were unable to do the stratified and interaction analysis by staging for liver cancer patients since there were scant number of liver cancer patients. However, we did estimate survival and hazard ratio for cholangiocarcinoma and head and neck cancer patients. We found similar phenomenon of the aHR for TB infection as in lung cancer. Therefore, the possible explanation for this would be similar as we hypothesized for lung cancer patients. However, the mechanism underlying these phenomena need to be investigated and clarified.

Regarding treatment, our result suggested that we should pay more attention to detection and treatment of TB infection in these three cancers types when comorbid with TB. Studies have unveiled that it is feasible to safely administer anti-tuberculosis and anti-cancer treatments, especially chemotherapy, simultaneously in lung and colorectal cancer patients with co-morbid TB (Kim et al., 2005; Hirashima et al., 2014; Chai and Shi, 2020). On the other hand, the comorbidity with TB in other types of cancer patients did not show any impacts on the survival time. Notably in our study, most of the patients were at late stage of cancers; therefore, symptomatic treatment was applied and may consider as no treatment needed. 

*Study limitations*

Firstly, 84.41% of patients had non-histological type of lung cancer who did not receive any treatments and 48.36% of patients who had histological type of lung cancer, which could lead to misclassification in our lung cancer patient cohort that did not have histological confirm. However, the overall survival of these two groups were comparable since 5-year- survival of patients with histological confirmation of lung cancer and those without histological confirmation was 7% and 8%, respectively. This reflects the similarity in the nature of underlying conditions of these diseases. Secondly, several related factors associated with the survival of cancer patients were not included in this study such as histological type, other comorbidities (diabetes or immunodeficiency), lifestyle, and family history of diseases. 

In conclusion, based-on our findings, it was found that lung, liver, and head and neck cancer patients with co-morbid TB had better survival than those without co-morbid TB. Co-morbid TB and stage of cancer could interact with and modify the risk of death for lung cancer patients. There is a need for further studies to support our findings including other related risk factors. 

## Author Contribution Statement

PS, SN, and PV were responsible for conceptualization and methodology. KS and CS contributed to data curation and investigation. PS and SN performed statistical analysis. PS and SN wrote the original draft. This study was supervised by PV and KS. PS, SN, and PV participated in editing the manuscript. All authors reviewed and approved the final version of the manuscript.

## Data Availability

The data that supported the findings of this study are available on request from the corresponding author, PV. The data are not publicly available since it contains information that can compromise the privacy of research participants.
